# Trunk and lower limb muscularity in sprinters: what are the specific muscles for superior sprint performance?

**DOI:** 10.1186/s13104-021-05487-x

**Published:** 2021-02-25

**Authors:** Nobuaki Tottori, Tadashi Suga, Yuto Miyake, Ryo Tsuchikane, Takahiro Tanaka, Masafumi Terada, Mitsuo Otsuka, Akinori Nagano, Satoshi Fujita, Tadao Isaka

**Affiliations:** 1 Research Organization of Science and Technology, Ritsumeikan University, 1-1-1 Nojihigashi, Kusatsu, 525-8577 Shiga Japan; 2 Faculty of Sport and Health Science, Ritsumeikan University, 1-1-1 Nojihigashi, Kusatsu, Shiga 525-8577 Japan

**Keywords:** Cross-sectional area, Joint torque, Hip flexion, Hip extension, Magnetic resonance imaging

## Abstract

**Objective:**

The major purpose of this study was to determine the specific muscle(s) for superior sprint performance in sprinters. The cross sectional areas (CSAs) of ten muscles of the trunk and lower limb were measured using magnetic resonance images in 56 male sprinters and 40 male non-sprinters. In addition to the absolute CSA, to minimize the effect of difference in body size among participants, the relative CSA normalized to body mass was used for analysis of this study.

**Results:**

Absolute and relative CSAs of most trunk and lower limb muscles, including the psoas major (PM) and gluteus maximus (GM), were significantly larger in sprinters than in non-sprinters (all *P* < 0.001, *d* = 0.91 to 1.82). The absolute and relative CSAs of the PM and GM correlated significantly with personal best 100-m sprint time in sprinters (*r* =  − 0.363 to − 0.388, all *P* < 0.01). A stepwise multiple regression analysis revealed that both CSAs of absolute PM and relative GM were predictive variables for the personal best 100 m sprint time in sprinters (*β* =  − 0.289 and − 0.287, respectively, both *P* < 0.05). These findings suggest that the PM and GM may be specific muscles for superior sprint performance in sprinters.

## Introduction

The generation of large torques by muscles crossing the hip, knee, and ankle joints is necessary for superior sprint performance in sprinters [1]. The joint torque is largely determined by size of agonist muscle group [2]. Previous studies have reported that many muscles of the trunk and lower limb were greater in sprinters than in non-sprinters [3–5]. Furthermore, previous studies have determined that the sizes of some trunk and lower limb muscles are correlated with superior sprint performance in sprinters [6–12]. However, many of these studies have examined using only a few muscles of the trunk and lower limb. Therefore, the specific muscles that contribute to superior sprint performance for sprinters have not been fully identified.

The contribution of the hip joint torque during sprinting is greater than that of the ankle and knee joint torques [1]; thus, greater hip muscles may play an important role for achieving better sprint performance in sprinters. The psoas major (PM) is a major muscle for the hip flexion. Several previous studies have reported that a greater PM size is correlated with better sprint performance in sprinters [7, 10, 12]. In addition, the gluteus maximus (GM) is a major muscle for the hip extensors. Sugisaki et al. reported that a greater GM size is correlated with better sprinter performance in sprinters [10]. However, the relationship between the GM size and sprint performance in sprinters remains poorly understood.

To gain our understanding of specific muscle(s) for superior sprint performance in sprinters, this study first examined the differences in cross-sectional areas (CSA) of 10 selected muscles of the trunk and lower limb between sprinters and non-sprinters. Thereafter, we examined the relationships between the trunk and lower limb muscle CSAs and sprint performance in sprinters.

## Main text

### Methods

#### Participants

Fifty-six well-trained male sprinters (age: 20.7 ± 1.6 years) participated in this study. Their personal best time of a 100 m race time ranged from 10.32 to 11.80 s (mean, 11.12 ± 0.36 s) within the previous one year. They were involved in regular sprint training at least 5 times per week and had regularly competition. In addition, 40 non-sprinters (age: 21.1 ± 1.1 years) whose body size (i.e., body height and body mass) was similar to those of the sprinters were selected as a control group (see Table 1). The body size-matched control participants were recreationally active, but were not involved in any specific physical training program within the previous 3 years. Nevertheless, many of them had participated in recreational sports and/or physical training for 2–3 h per week. All participants were informed of the experimental procedures and provided written consent to participate in the study. This study was approved by the Ethics Committee of Ritsumeikan University.

#### Magnetic resonance imaging (MRI)

**Fig. 1:**
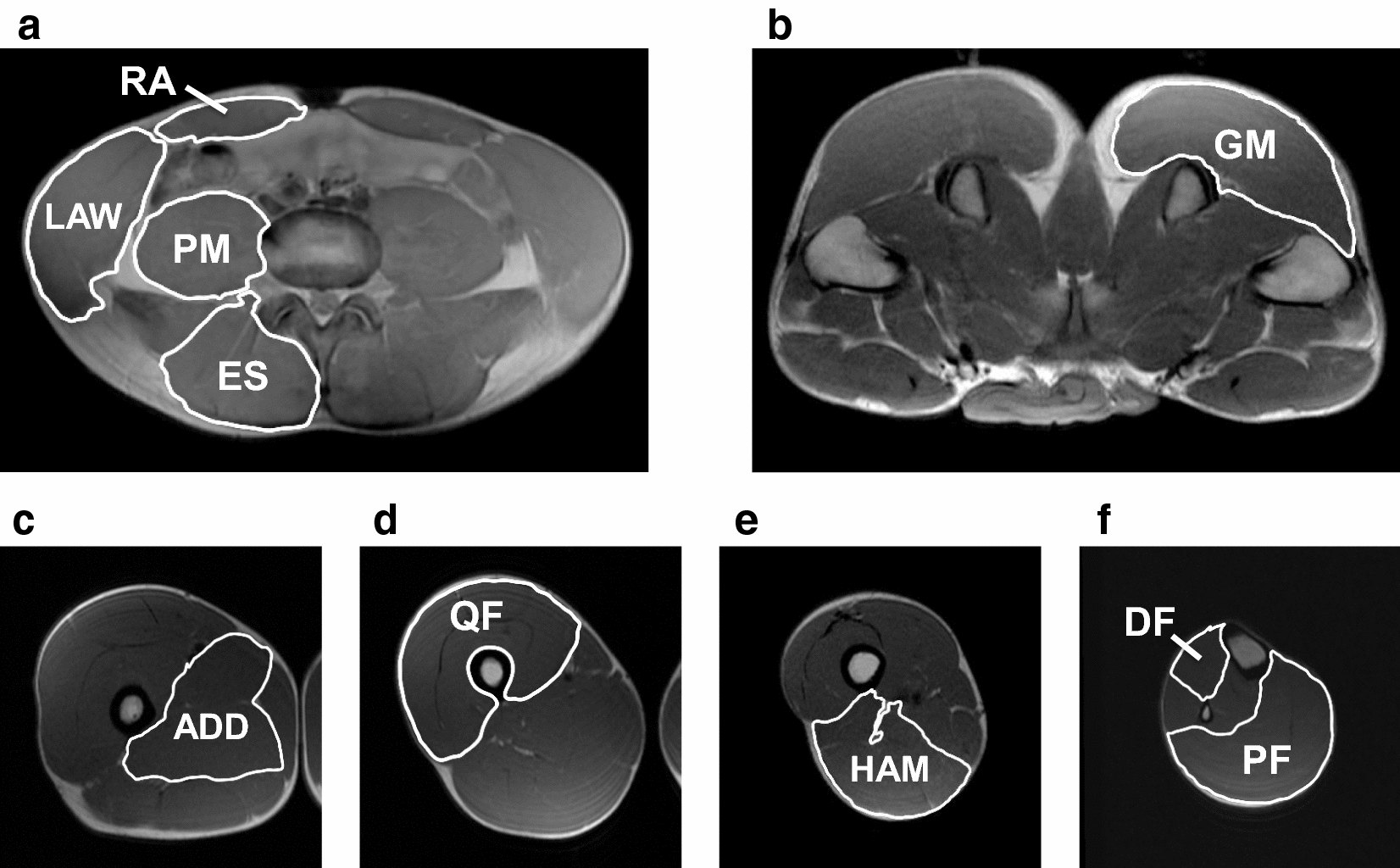
Representative magnetic resonance imaging scans for measuring cross-sectional areas of trunk and lower limb muscles. The cross-sectional areas (CSA; CSAs) of the rectus abdominis (RA), lateral abdominal wall (LAW), elector spinae (ES), and psoas major (PM) were obtained at the mid-level of the L4–L5. **a** The CSA of the gluteus major (GM) was obtained at the level of the greater trochanter. **b** The CSA of the adductors (ADD) was obtained at the proximal 30% of the thigh length. **c** The CSA of the quadriceps femoris (QF) was obtained at 50% of the thigh length. **d** The CSA of the hamstrings (HAM) was obtained at 70% of the thigh length. **e** The CSAs of the dorsiflexors (DF) and plantar flexors (PF) were obtained at the proximal 30% of the lower leg length (**e**)

Representative MRI scans for measuring CSAs of the trunk and lower limb muscles are presented in Fig. 1. To avoid any effects from changes in muscle size due to heavy training, MRI measurements for sprinters were scheduled the following day after a rest day or light intensity training day during the off-season. Additionally, MRI measurements for sprinters who performed the light intensity training were scheduled at least 12 h after the training session. The MRI measurement has been described in our previous studies [11–13]. In brief, to measure the CSAs of the trunk and lower limb muscles, excluding the GM, participants were placed in a supine position on the scanner bed, with both knees fully extended and both ankles set at the neutral position (i.e., 0°). With regard to the trunk muscles, the CSAs of the rectus abdominis, lateral abdominal wall, erector spinae, and PM were obtained at the mid-level of the L4-L5 [7, 9, 11, 12]. With regard to the lower limb muscles, the CSAs of the adductors, quadriceps femoris (QF), hamstring (HAM), dorsiflexors (DF), and plantar flexors were measured. The adductors CSA was obtained at the proximal 30% of the thigh length [9]. The QF CSA was obtained at the proximal 50% of the thigh length [9, 13]. The HAM CSA was obtained at the proximal 70% of the thigh length [9]. The DF and plantar flexors CSAs were obtained at the proximal 30% of the lower leg length [9]. To measure the CSA of the GM, participants were placed in a prone position on the scanner, with both knees fully extended. The GM CSA was obtained at the level of the greater trochanter. The locations for calculating these trunk and lower limb CSAs were based on methods outlined in our and other previous studies [7, 9, 11–13].

The CSAs of all ten muscles were calculated from the right side using image analysis software (OsiriX Version 5.6, Switzerland). In addition to the absolute CSA, to minimize the effects of difference in body size among participants, the relative CSA normalized to body mass to the two-thirds power was used for analysis of this study, as in our and other previous studies [9, 13].

### Statistical analysis

Data are expressed as mean ± SD. Comparisons of measured variables between sprinters and non-sprinters were performed using unpaired *t*-testing. The Cohen’s *d* effect size using the pooled SD was calculated to determine the magnitude of difference in outcome variables between the two groups. The magnitudes of the effect size were interpreted as small (0.20 ≤ *d* < 0.50), medium (0.50 ≤ *d* < 0.80) and large (0.80 ≤ *d*) [14]. The relationships between trunk and lower limb muscle CSAs and personal best 100-m sprint time in sprinters were evaluated using a Pearson’s product moment correlation coefficient. A stepwise multiple regression analysis was used to determine the predictive variable(s) for the personal best 100-m sprint time in sprinters using the absolute and relative CSAs as independent variables. Statistical significance level was defined at *P* < 0.05. All statistical analyses were conducted using the IBM SPSS software (version 19.0; International Business Machines Corp, NY, USA).

## Results

Physical characteristics and absolute and relative CSAs of the trunk and lower limb muscles in sprinters and non-sprinters are listed in Table 1. Body height and body mass did not differ significantly between sprinters and non-sprinters. The absolute and relative CSAs of most trunk and lower limb muscles, excluding the QF and DF, were significantly larger in sprinters than in non-sprinters (all *P* < 0.001), with large effect size (*d* = 0.91 to 1.82).

**Table 1:** Physical characteristics and absolute and relative cross-sectional areas (CSA) of trunk and lower limb muscles in sprinters and non-sprinters

	Sprinters	Non-sprinters	*P* value	Cohen’s *d*
Body height, cm	175.4 ± 5.1 [174.0, 176.8]	174.6 ± 4.7 [173.2, 176.0]	0.613	0.16
Body mass, kg	66.0 ± 5.1 [64.6, 67.4]	66.4 ± 7.2 [64.1, 68.6]	0.798	0.06
Absolute CSA, cm^2^				
Rectus abdominis	8.7 ± 1.7 [8.2, 9.1]	6.8 ± 1.8 [6.2, 7.3]	< **0.001**	**1.13**
Lateral abdominal wall	28.3 ± 4.8 [27.0, 29.6]	23.5 ± 4.0 [22.3, 24.7]	< **0.001**	**1.07**
Erector spinae	25.4 ± 3.3 [24.5, 26.3]	22.3 ± 3.7 [21.1, 23.4]	< **0.001**	**0.91**
Psoas major	20.5 ± 2.7 [19.8, 21.3]	16.9 ± 2.5 [16.1, 17.6]	< **0.001**	**1.40**
Gluteus maximus	60.1 ± 7.2 [58.2, 62.0]	50.8 ± 5.8 [49.0, 52.6]	< **0.001**	**1.41**
Quadriceps femoris	77.1 ± 8.2 [74.9, 79.3]	75.6 ± 10.7 [72.2, 78.9]	0.427	0.16
Hamstring	46.8 ± 6.7 [45.0, 48.6]	37.7 ± 7.2 [35.5, 40.0]	< **0.001**	**1.31**
Adductors	58.2 ± 6.3 [56.5, 59.9]	49.4 ± 8.4 [46.8, 52.0]	< **0.001**	**1.22**
Dorsiflexors	10.9 ± 1.2 [10.6, 11.2]	10.6 ± 1.5 [10.2, 11.1]	0.367	0.20
Plantar flexors	50.5 ± 7.2 [48.5, 52.4]	44.3 ± 5.9 [42.5, 46.1]	< **0.001**	**0.92**
Relative CSA, cm^2^/kg^2/3^				
Rectus abdominis	0.53 ± 0.09 [0.51, 0.56]	0.41 ± 0.10 [0.38, 0.44]	< **0.001**	**1.26**
Lateral abdominal wall	1.73 ± 0.27 [1.66, 1.80]	1.43 ± 0.21 [1.37, 1.50]	< **0.001**	**1.21**
Erector spinae	1.56 ± 0.18 [1.51, 1.60]	1.36 ± 0.18 [1.30, 1.42]	< **0.001**	**1.10**
Psoas major	1.26 ± 0.15 [1.22, 1.30]	1.03 ± 0.14 [0.99, 1.07]	< **0.001**	**1.58**
Gluteus maximus	3.68 ± 0.33 [3.59, 3.76]	3.10 ± 0.30 [3.01, 3.19]	< **0.001**	**1.82**
Quadriceps femoris	4.72 ± 0.42 [4.61, 4.83]	4.60 ± 0.45 [4.46, 4.74]	0.192	0.27
Hamstring	2.86 ± 0.36 [2.77, 2.96]	2.29 ± 0.34 [2.19, 2.40]	< **0.001**	**1.62**
Adductors	3.56 ± 0.31 [3.48, 3.65]	3.01 ± 0.41 [2.89, 3.14]	< **0.001**	**1.55**
Dorsiflexors	0.67 ± 0.07 [0.65, 0.69]	0.65 ± 0.09 [0.62, 0.68]	0.291	0.24
Plantar flexors	3.09 ± 0.37 [2.98, 3.19]	2.70 ± 0.29 [2.61, 2.79]	< **0.001**	**1.12**

Values are presented as Mean ± SD [the upper and lower limits of a 95% confidence interval]. The relative CSA was normalized to body mass to the two-thirds power. Bold values indicate significance differences (*P* < 0.05) between sprinters and non-sprinters

Correlation coefficients of absolute and relative CSAs of the trunk and lower limb muscles with personal best 100-m sprint time in sprinters are shown in Table 2. Among the ten muscles, the absolute and relative CSAs of only the PM and GM correlated significantly with personal best 100-m sprint time in sprinters (*r* =  − 0.363 to − 0.388, all *P* < 0.01). A stepwise multiple regression analysis revealed that both CSAs of absolute PM and relative GM were predictive variables for the personal best 100-m sprint time in sprinters (*β* =  − 0.289 and − 0.287, respectively, both *P* < 0.05). The adjusted *R*^2^ for this analysis was 0.194 (*P* = 0.001).

**Table 2:** Correlation coefficients between absolute and relative CSAs of trunk and lower limb muscles and personal best 100-m sprint time in sprinters

	*r* [the upper and lower limit of a 95% CI]	*P* value
Absolute CSA		
Rectus abdominis	− 0.009 [− 0.271, 0.255]	0.945
Lateral abdominal wall	− 0.200 [− 0.440, 0.066]	0.140
Erector spinae	− 0.119 [− 0.149, 0.370]	0.383
Psoas major	− **0.388 [**− **0.591, **− **0.139]**	**0.003**
Gluteus maximus	− *0.366 [*− *0.574, *− *0.114]*	**0.006**
Quadriceps femoris	− 0.040 [− 0.300, 0.225]	0.767
Hamstring	− 0.107 [− 0.360, 0.160]	0.434
Adductors	− 0.092 [− 0.347, 0.175]	0.502
Dorsiflexors	0.088 [− 0.179, 0.343]	0.521
Plantar flexors	− 0.132 [− 0.382, 0.136]	0.332
Relative CSA		
Rectus abdominis	0.041 [− 0.224, 0.301]	0.761
Lateral abdominal wall	− 0.169 [− 0.414, 0.098]	0.213
Erector spinae	− 0.064 [− 0.321, 0.202]	0.639
Psoas major	− **0.363 [**− **0.571, **− **0.111]**	**0.006**
Gluteus maximus	− **0.387 [**− **0.590, **− **0.138]**	**0.003**
Quadriceps femoris	0.040 [− 0.225, 0.300]	0.769
Hamstring	− 0.056 [− 0.314, 0.210]	0.679
Adductors	− 0.020 [− 0.281, 0.244]	0.884
Dorsiflexors	0.170 [− 0.097, 0.414]	0.209
Plantar flexors	− 0.082 [− 0.338, 0.185]	0.548

Bold values indicate significant correlations (*P* < 0.05) between CSA variables and personal best 100-m sprint time

## Discussion

This study determined that the absolute and relative CSAs of most trunk and lower limb muscles, excluding the QF and DF, were larger in sprinters than in non-sprinters. In particular, absolute CSAs of the PM and GM was 21.7 and 18.4%, respectively, higher in sprinters than in non-sprinters. In addition, the primary findings of this study showed that larger absolute and relative CSAs of the PM and GM correlated with better personal best 100 m sprint time in sprinters. Furthermore, a stepwise multiple regression analysis revealed that both CSAs of absolute PM and the relative GM were predictive variables of the personal best 100-m sprint time. These findings suggest that the PM and GM may be specific muscles for superior sprint performance for sprinters.

With regard to the PM, it is known to be a major muscle for performing hip flexion because of the largest among the hip flexors [15]. Hoshikawa et al. [16] reported that larger PM CSA is correlated with higher hip flexor maximal torque. Moreover, using a computer simulation, Dorn et al. [17] determined that the PM (i.e., which combined the psoas major and iliacus) was the highest torque component for performing the hip flexion and contributed to rapidly accelerating the leg forward during sprinting. Furthermore, we and others previously reported that larger PM CSA is correlated with better sprint performance (e.g., personal best 100-m sprint time) in sprinters [7, 10–12]. Therefore, the present finding corroborates the results of these previous studies by showing positive correlations between absolute and relative PM CSAs and personal best 100-m sprint time in sprinters. The findings of our and other studies suggest that the PM is an important muscle for achieving superior sprint performance in sprinters.

With regard to the GM, it is known to be a major muscle for performing hip extension because of the largest among the hip extensors [4, 18]. Tayashiki et al. [19] reported a positive correlation between the GM thickness and hip extensor maximal torque, but this correlation did not reach significance. Moreover, Bartlett et al. [18] determined that an increase in the electromyographic activity of the GM is related to increased sprint velocity when sprinting. In addition, the GM plays an important role in decelerating the forward swing of the leg during the latter half of the swing phase and stabilizing the trunk against flexion while sprinting [17, 20]. Furthermore, the computer simulation study, by Dorn et al. [17], determined that an increase in step frequency from a slow running to maximal sprinting is principally achieved by increasing the work of the hip extensor muscles, especially the GM, during the latter half of the swing phase. The increased step frequency is necessary to increasing sprint velocity during spirting [12, 21, 22]; thus, larger GM plays an important role in achieving better sprint performance, potentially by enhancing step frequency. Despite these previous findings, the relationship between GM size and sprint performance in sprinters remains poorly understood. Sugisaki et al. determined that greater muscle volume (MV) of the GM is correlated with better personal best 100 m sprint time in sprinters [10]. Therefore, the present finding corroborates their result by showing positive correlations between the absolute and relative GM CSAs and personal best 100 m sprint time in sprinters. Altogether, in addition to the PM, the GM is an important muscle for achieving superior sprint performance in sprinters.

In addition to the GM, the HAM, which is a biarticular muscle that extends the hip and flexes the knee, is known to be another major muscle among the hip extensors. The HAM is often considered a key muscle for achieving superior sprint performance in sprinters [23]. However, we and others previously reported the absence of relationship between the HAM CSA and sprint performance (e.g., personal best 100 m sprint time) in both groups of junior and adults sprinters [7, 9, 12]. Therefore, the present finding corroborates the results of these previous studies by showing no correlations between the absolute and relative HAM CSAs and personal best 100-m sprint time in adult sprinters. The findings of our and other studies suggest that the HAM may not be an important muscle for achieving superior sprint performance in sprinters.

## Limitations

Although we used the CSAs for evaluating muscle size, it has been considered that the MV is a more reliable marker of muscle size than CSA [2]. Moreover, although we measured the CSAs of 10 selected muscles of the trunk and lower limb, number of muscles included in this study was relatively low when compared to that of several previous studies [4, 10]. Furthermore, although we selected the PM and QF (i.e., the rectus femoris) as the hip flexors, it includes other agonist muscles such as the iliacus. Similarly, although we selected the GM and HAM as the hip extensors, it includes other synergistic muscles such as the gluteus minimus and gluteus medius. To further clarify the findings of the present study, further studies are needed to examine the relationships between MVs of all hip flexor and extensor muscles and sprint performance in sprinters.

## Data Availability

Data will be provided the corresponding author upon request.

## Abbreviations

CSA: Cross-sectional area
DF: Dorsiflexors
GM: Gluteus maximus
HAM: Hamstring
MRI: Magnetic resonance imaging
MV: Muscle volume
PM: Psoas major
QF: Quadriceps femoris
